# Preliminary Evaluation of Two iiPCR Platforms for Qualitative Field Detection of *Cannabis sativa*

**DOI:** 10.3390/plants15193056

**Published:** 2026-10-07

**Authors:** Sydney Hampton, Greta Hundtofte, Rachel Houston

**Affiliations:** Department of Forensic Science, College of Criminal Justice, Sam Houston State University, Huntsville, TX 77341, USA; smh168@shsu.edu (S.H.); geh032@shsu.edu (G.H.)

**Keywords:** forensic plant science, hemp, marijuana, field-deployable

## Abstract

Insulated isothermal PCR (iiPCR) amplifies DNA through convection-based thermocycling and is therefore well suited for field-deployable screening. Here, a commercial iiPCR cannabis assay was evaluated on a benchtop platform (POCKIT Central) and a field-portable platform (POCKIT Micro Duo). The assay targets cannabis DNA rather than THC content and therefore identifies hemp and marijuana alike, matching jurisdictions that control all cannabis material regardless of THC content. Sensitivity, exclusivity, variety, and interference studies were performed under controlled laboratory conditions. The assay detected cannabis in all 31 hemp powder varieties, five marijuana samples, and five commercial hemp seed samples tested, as well as in cannabis–tobacco mixtures containing up to a 1000-fold excess of tobacco. Of twelve non-cannabis materials tested, only hops (*Humulus lupulus*) returned a positive result, on one of four replicates using the benchtop platform. The benchtop system demonstrated greater sensitivity than the portable platform when using the assay. Both platforms returned results within 85 min without laboratory infrastructure, supporting screening of plant material and reducing the number of samples that require confirmatory analysis.

## 1. Introduction

*Cannabis sativa* L., a member of the family Cannabaceae [1], is cultivated to produce both marijuana and hemp, which are commonly differentiated based on their tetrahydrocannabinol (THC) content. Hemp, which contains little to no THC relative to marijuana, is frequently utilized in the manufacture of products such as fibers [2] or for therapeutic applications [3], whereas marijuana is primarily consumed recreationally.

Despite its long history of use [4], the legality of cannabis possession, distribution, and consumption varies widely due to its psychoactive effects. In the United States of America, cannabis plant material classified as hemp must contain no more than 0.3% delta-9 THC on a dry weight basis; material exceeding this threshold is classified as marijuana and is considered federally illicit under the Agriculture Improvement Act of 2018 [5]. In contrast, several countries enforce strict regulatory frameworks that classify cannabis, including both hemp and marijuana, as a controlled substance. Taiwan classifies cannabis as an illegal substance [6], and Indonesia classifies all parts of the cannabis plant, including processed materials, as a group I controlled substance, prohibiting its use for medical applications [7].

Despite differences in legal status across jurisdictions, cannabis products continue to be encountered in illicit importation and exportation. Notably, cannabis has been hidden in statues [8] or manifested as chocolate [9]. While current approaches for detecting smuggled drug materials, such as trained canines [10] and X-ray devices [8], can indicate the presence of suspected materials, they do not provide identification of the material itself. Therefore, selective and sensitive methods that are not influenced by the presence of additional materials are required for presumptive identification.

Forensic laboratories continue to experience significant evidence backlogs [11], emphasizing the need for improved field-based presumptive testing prior to confirmatory laboratory analysis. Field-based or presumptive identification of cannabis materials commonly includes colorimetric tests [12] and portable spectroscopic instruments [13]. Colorimetric tests can be performed in situ with minimal training but rely on subjective interpretation, increasing the potential for false positive and false negative results. Portable spectroscopic techniques, such as Fourier transform infrared (FTIR) and Raman spectroscopy, reduce subjectivity through spectral library comparisons. However, FTIR analysis of plant material is often complicated by the presence of water, which can obscure spectral features and hinder clear identification [14]. Generally, Raman spectroscopy is less suitable for the analysis of complex mixtures, especially when the mixture components have spectral features that overlap and require more complex data analysis [15]. While the use of gas chromatography-mass spectrometry (GC-MS) is the gold standard method to determine if cannabinoids are present in samples, commercial portable gas chromatographs that could be used for presumptive identification are not commonly used and require training for field applications. As existing screening techniques have limitations for the presumptive detection of cannabis materials, integration of biological methods into drug screening workflows may provide an efficient and sensitive approach for the identification of naturally derived materials.

Insulated isothermal PCR (iiPCR) is a promising approach for field-based cannabis detection because it amplifies DNA within a single reaction vessel without a conventional thermocycler. The instrument instead heats only the base of the tube and holds it at a constant temperature, so that the bottom of the reaction remains near denaturation temperature while the top remains cooler, and thermal convection carries reagents between these zones [16]. As in conventional PCR, the target region is chosen for specificity to the organism of interest, and amplification is monitored by a fluorescent probe. The assays evaluated here report a presence or absence call against a fluorescence threshold rather than a quantitative value. Reported limits of detection are low, with one iiPCR method identifying samples containing as few as 100 copies of the target DNA [17].

This approach can be implemented using both benchtop and field-portable instruments, including POCKIT Central Nucleic Acid Analyzer and POCKIT Micro Duo Nucleic Acid Analyzer (GeneReach Biotechnology Corp., Taichung, Taiwan). According to the manufacturer, these instrument designs are intended for operation by minimally trained analysts and provide multiple-sample throughput and rapid turnaround, enabling screening outside of laboratory environments such as at ports of entry and airports.

To date, published iiPCR applications in plants have focused on the detection of organisms associated with plants, rather than utilizing iiPCR for identification of the plant material itself. Previously, iiPCR has been applied to the detection of fungi, α-proteobacteria, and pathogens such as *Fusarium oxysporum* [18], *Candidatus* Liberibacter asiaticus [19], and *Acidovorax citrulli* [20], respectively.

In this study, the evaluation of POCKIT Central and POCKIT Micro Duo was completed using the GeneReach cannabis assay. The objective was to determine whether either platform provides consistent presumptive identification of cannabis across commercially available plant materials, and to characterize where their detection limits and cross-reactivity lie. The evaluation included sensitivity, exclusivity, variety, and interference studies, as well as marijuana and hemp seed samples. We hypothesized that both platforms would detect cannabis DNA across hemp varieties, marijuana, and non-leaf tissue irrespective of THC content and in the presence of excess cutting agents, and that any cross-reactivity would be confined to close relatives within the Cannabaceae family rather than to unrelated botanical materials. We further expected the benchtop platform, which performs a full extraction, to detect cannabis at lower template concentrations than the portable platform, which relies on a single-step crude lysis.

## 2. Results and Discussion

### 2.1. Sensitivity Study

The POCKIT Central was the more sensitive platform (Table 1), detecting cannabis DNA in one of three replicates at the 10^−4^ dilution, at which the POCKIT Micro Duo gave no detections. Across the series, detection in every replicate extended two orders of magnitude further on the POCKIT Central than on the POCKIT Micro Duo.

Across the dilution series, the POCKIT Central returned a positive result in 17 of 25 reactions and the POCKIT Micro Duo in 8 of 25. Because reactions on both platforms were drawn from the same dilution tubes, template input was matched, and this difference is attributable to the platforms rather than to sample preparation.

The inconsistency observed at the lower dilutions of the sensitivity series (Table 1) reflects how the assays report results. Each returns a presence or absence call against a fixed fluorescence threshold applied to the proprietary target region. When DNA quantities are near the assay threshold, resulting fluorescence may also approach the detection limit, creating inconsistency in DNA detection and reporting. This accounts for the non-monotonic pattern seen on the POCKIT Micro Duo, where detection occurred at some dilutions more extreme than others that gave none; such reversals are expected once template copy number approaches one per reaction. The difference between the two platforms, by contrast, is more likely to originate in sample preparation. The POCKIT Central extracts DNA using two wash buffers and magnetic beads before the extract is added to the R-tube, whereas the POCKIT Micro Duo relies on a single-step crude lysis. It is therefore likely that the full extraction method of the Central recovers more amplifiable DNA per reaction, accounting for its greater sensitivity.

In practical terms, sensitivity here is expressed relative to the extract prepared from a single sampling of approximately 5 mg of ground material. Because the dilution series was prepared from crude extract without DNA quantification, the limit of detection is reported here in dilution terms rather than as a copy number. Consistent detection at a thousand-fold dilution on the POCKIT Central therefore corresponds to roughly 5 µg of starting plant material, well below the quantity present in typical seized plant material. Despite detecting across a narrower range, the POCKIT Micro Duo successfully identified cannabis in every undiluted sample tested in this study. The lower sensitivity observed for the portable platform is unlikely to affect field screening of bulk materials, as these samples are expected to contain sufficient quantities of DNA.

The replicate numbers used in this study are insufficient to resolve detection probability near the limit of detection, and the non-monotonic pattern observed on the POCKIT Micro Duo at the 10^−3^ and 10^−4^ dilutions reflects imprecision rather than a property of the assay. The limit of detection is therefore presented as the lowest dilution at which detection was consistent rather than as a definitive threshold.

### 2.2. Exclusivity Study

As a screening method for cannabis materials, it is critical that non-cannabis-containing samples will have accurate results using the iiPCR methods. For this purpose, naturally derived materials and human DNA were assessed to determine the potential for cross-reactivity using the iiPCR methods.

As shown in Table 2, all the non-cannabis materials were negative for cannabis DNA for both the POCKIT Central and the POCKIT Micro Duo (12/12, 100%, 95% CI 75.7–100%). On the POCKIT Central, 11 of the 12 were negative (91.7%, 95% CI 64.6–98.5%); hops returned a positive result in one of four replicates.

Negative results for the non-cannabis materials would suggest that these materials can be excluded from requiring confirmatory identification of cannabis. For this reason, this assessment is referred to as an exclusivity study. Because this was the only positive obtained from a non-cannabis material, additional replicates of hops were assessed on both the portable and benchtop platforms. Inconsistency in the results from hops samples is likely due to the close relation between cannabis and hops, as they share the same family, Cannabaceae [21]. Beyond shared taxonomy, the two genera exhibit some overlapping features, such as serrated leaf architecture [22,23]. However, once these plant materials are removed for processing into commercial goods, differentiating them can be much more challenging, as morphological characteristics often cannot be used for differentiation. Further, as previously explained, the POCKIT Central is more sensitive than the POCKIT Micro Duo and would therefore be more likely to amplify low-abundance hops sequences that resemble the cannabis target region.

Cross-reactivity with closely related botanicals is not unique to DNA-based screening; the presumptive tests currently in routine use carry comparable specificity limitations. Color tests remain the standard presumptive approach in seized-drug screening. For cannabis, the Duquenois–Levine (D-L) test is the established presumptive identification, while 4-aminophenol (4-AP) and Fast Blue BB (FBBB) differentiate hemp and marijuana [24]. Their reliability, however, is constrained by several well-documented limitations. The D-L test has been shown to yield false positives for eucalyptus and patchouli [25]. Prior work on the same test also indicates that solid samples must be extracted before the color test is run; omitting this step can lead to false reports [12]. Although the 4-AP and FBBB tests have been proposed as a paired approach for differentiating hemp from marijuana, they return inconclusive results for samples with comparable THC and cannabidiol (CBD) content [24].

By contrast, the portable and benchtop iiPCR platforms evaluated here incorporate an internal control and standardized extraction workflow across various sample types, providing users with means of verifying successful assay performance. Combining these features could support the rapid and objective screening of cannabis materials in field-based settings, reducing false and inconclusive results.

### 2.3. Variety Studies

Across the hemp variety, marijuana, and hemp seed studies, all 41 cannabis materials were detected on both platforms (41/41, 100%, 95% CI 91.4 to 100%).

#### 2.3.1. Hemp Variety Study

The use of different cultivation methods has resulted in variations in cannabis plant composition [26,27], allowing growers to select for specific traits within cultivated materials. Therefore, analysis of diverse hemp materials obtained from multiple sources was necessary to evaluate potential variability among samples for the two iiPCR methods discussed. All 31 hemp varieties tested were correctly identified as cannabis on both the POCKIT Central and the POCKIT Micro Duo, with one replicate per variety on each instrument.

#### 2.3.2. Marijuana Study

The iiPCR methods described here detect cannabis DNA but do not differentiate hemp from marijuana; accordingly, samples classified as marijuana were evaluated in addition to the hemp materials described above. All five marijuana samples were positive for cannabis in both replicates (2/2) on both the POCKIT Central and the POCKIT Micro Duo.

#### 2.3.3. Hemp Seed Study

Hemp-derived products sold in ordinary grocery channels, rather than in smoke shops, are among the most widely available cannabis materials to the public. Hemp seeds were therefore selected to determine whether the assay could detect cannabis in plant tissue other than leaf material. All five hemp seed samples were correctly identified as cannabis on both the benchtop POCKIT Central and the portable POCKIT Micro Duo (1/1 replicate per sample on each instrument).

### 2.4. Interference Study

Tobacco is a common cutting agent for hemp products [28,29]. For this reason, increasing amounts of tobacco were combined with cannabis material to produce the ratios listed in Table 3. The POCKIT Central was evaluated at 1×, 10×, 50×, 100×, 200×, and 1000×, while the less sensitive POCKIT Micro Duo was evaluated at 1×, 5×, and 10×. Cannabis was detected at every ratio tested on each instrument.

### 2.5. Limitations and Future Directions

Recently, two different types of assays, a TaqMan™ quantitative PCR assay and a Capillary Electrophoresis (CE)-based SNaPshot™ assay, were compared for the differentiation of hemp and marijuana, and it was found that the TaqMan™ quantitative PCR assay was more efficient [30]. Although these PCR-based assays are effective in determining the presence of cannabis and differentiating hemp from marijuana, sample preparation and analysis are often costly, require specialized training, and are not amenable to rapid processing, limiting their suitability for routine screening of seized drug samples. Furthermore, these techniques are not readily portable, precluding their use for in-field analysis.

The use of these iiPCR methods can allow for the identification of cannabis by people without extensive and costly training outside of classical forensic DNA or seized drug laboratories.

The benchtop instrument, POCKIT Central, requires an electrical outlet to run but can perform DNA extractions before analyzing the samples. The handheld and portable instrument, POCKIT Micro Duo, has a rechargeable battery but requires a separate crude lysis step before analysis. The decision of which instrument to use when applying this research will depend on the environment where samples are being tested. In this study, the same sample types were assessed using both the benchtop and portable instruments to allow the direct comparison of the results and evaluation of the capabilities of each platform.

The crude extracts were not quantified, so the limit of detection is expressed as a dilution of the extract rather than as a copy number. Replicate depth was limited, as described in Section 3.1.1, and the sensitivity study in particular cannot resolve detection probability near that limit. Comparisons against colorimetric and spectroscopic presumptive tests rest on the published performance of those methods, as no parallel testing was performed here. As this is strictly a screening technique, samples that test positive for cannabis DNA would require confirmatory testing. However, the elimination of more complex sample analysis for non-cannabis materials can assist in the decrease in forensic backlog of evidence.

A second set of constraints follows from the proprietary composition of the commercial assay. Because the target region and primer sequences are not published, cross-reactivity cannot be predicted in silico and can be identified only empirically; a negative result on a divergent variety cannot be attributed to primer mismatch rather than to low template, and the copy number of the target is unknown. Results are specific to the assay version tested, as a proprietary formulation may be revised without notice. The assay is nonetheless commercially available, so the findings reported here can be reproduced and extended by other laboratories. Detection in all 31 hemp varieties, five marijuana samples, and five hemp seed products provides functional evidence of target conservation across the materials assessed, while the single-hop positive indicates that cross-amplification within the Cannabaceae cannot be excluded.

All testing was conducted under controlled laboratory conditions. Future work could include direct comparison against established presumptive tests, a broader range of commercially available cannabis products, and samples collected and field tested at ports of entry.

## 3. Materials and Methods

### 3.1. Sample and Reaction Preparation

#### 3.1.1. Sample Mass

**Figure A1:**
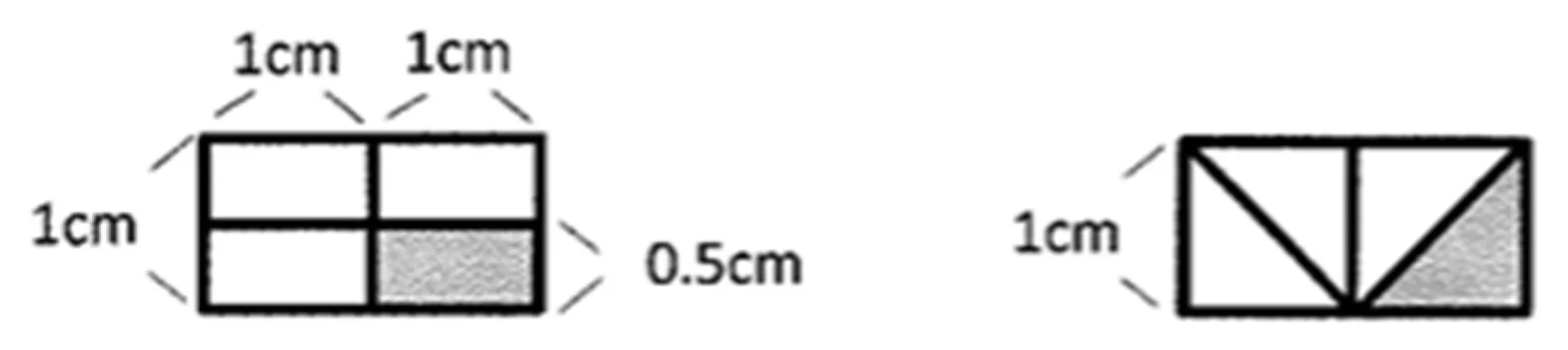
Sampling scheme provided by GeneReach Biotechnology Corp. [32]. The shaded (gray) region indicates the area of the template to be filled with sample material, corresponding to approximately 5 mg.

Both the benchtop POCKIT Central Nucleic Acid Analyzer and the portable POCKIT Micro Duo Nucleic Acid Analyzer use a sampling template supplied by the manufacturer to standardize the mass of sample added to each reaction under field conditions (Figure A1). Small fragments of samples were spread to fill the shaded region of the template, which corresponds to approximately 5 mg. Unless otherwise specified, Jazzy CBG (CBD Hemp Direct, Las Vegas, NV, USA) was used in each of the following studies, and every sample was analyzed on both platforms. Replicate numbers throughout were chosen to prioritize breadth of material coverage over replicate depth per sample, as the assay returns a binary presence or absence call rather than a measured value.

#### 3.1.2. Reaction Tubes and Premix

Both the POCKIT Central and POCKIT Micro Duo utilize R-tubes that are manufactured to allow for a temperature gradient from the bottom of the tube, where a copper ring is located, to the top of the tube. This gradient allows a cyclic flow of replication of the DNA sample, with denaturation occurring near the bottom of the tube, extension in the center of the tube, and annealing near the top [16]. Both assays supply the required reagents as a lyophilized premix, but the two platforms rehydrate it differently. The POCKIT Central requires only the premix tube to complete the reaction cartridge, whereas the POCKIT Micro Duo requires a pre-portioned volume of premix buffer to be added to the premix tube. Each premix also contains an internal control that is amplified in parallel with the cannabis target and reported alongside it, so that a negative cannabis result is distinguishable from a failed reaction.

#### 3.1.3. POCKIT Central

The POCKIT Central uses a proprietary buffer solution in a Sample Storage Solution (3S Tube) to prepare the material for DNA extraction. Cannabis material or other plant material was ground in the 3S Tube for approximately 1 min using a plastic pestle, and 200 µL of the resulting suspension was transferred to the sample cartridge. The POCKIT Central processes up to eight samples per run, with extraction, amplification, and analysis completed in 85 min. Prior to sample testing, the platform was verified using the positive control provided with the assay kit and a negative control consisting of a 3S Tube processed without analyte.

#### 3.1.4. POCKIT Micro Duo

For the POCKIT Micro Duo, crude extracts were prepared using Grind-N-Go DNA Extraction Sets, in which plant material is ground directly into a proprietary buffer solution. For each reaction, 1 µL of the resulting crude extract was transferred to an R-tube and combined with the rehydrated premix. The POCKIT Micro Duo processes four samples per run, with amplification and analysis completed in 42 min. Prior to sample testing, the platform was verified using a no-template negative control in which nuclease-free water was substituted for the crude extract.

#### 3.1.5. Proprietary Assay Components and Conditions for Replication

The cannabis assay evaluated here is supplied as a closed commercial system. The oligonucleotide primer and probe sequences, the target locus, the amplicon size, the composition of the lyophilized premix, the identity of the internal control, and the composition of the Sample Storage Solution (3S) and Grind-N-Go extraction buffers are proprietary to GeneReach Biotechnology Corporation (Taichung, Taiwan) and were not disclosed to the authors at any stage of the study. The authors are therefore unable to report these parameters, and no attempt was made to determine them by sequencing or other characterization of the reagents. Reaction conditions are fixed in the instrument firmware and are not user-configurable: the operator selects the assay, and the platform executes a preset amplification and detection program without exposing the temperature profile, cycle parameters, or fluorescence thresholds. Replication of the experiments reported here consequently requires the specific instruments, cartridges, and reagent lots identified below and cannot be achieved with open reagents or a generic PCR platform. To allow the materials used to be obtained and the results independently verified, the following identifiers are reported: POCKIT Central Nucleic Acid Analyzer (catalogue no. apcc, software/firmware version V2.2-220411); POCKIT Micro Duo Nucleic Acid Analyzer (catalogue no. apcm.3, software/firmware version V1.1-171228); POCKIT Central cannabis assay premix (catalogue no. a05005, lot no. CsC0806247); POCKIT Micro Duo cannabis PCR reagent set (catalogue no. a04004, lot no. 080624); R-tubes (catalogue no. b-rtube-48, lot no. 040324 and 040225); Sample Storage Solution 3S tubes (catalogue no. atc-lysis2, lot no. 092721 and 102521); Grind-N-Go DNA Extraction Sets (catalogue no. agng-dna, lot no. 031224 and 111824); and the sampling template (catalogue no. a04004), all supplied by GeneReach Biotechnology Corporation.

### 3.2. Sensitivity Study

In this study, sensitivity refers to the lowest concentration of cannabis DNA that can be detected by the assay. For POCKIT Central analysis, a full-concentration sample was prepared from one shaded region of Jazzy CBG as described in Section 3.1.3. Serial dilutions of 10^−1^, 10^−2^, 10^−3^, 10^−4^, 10^−5^, 10^−6^, 1/333, and 1/3333 were then created using the full-concentration sample and deionized water. The full-concentration and 10-1 dilutions were analyzed in two replicates; all remaining dilutions were analyzed in three replicates. For POCKIT Micro Duo analysis, a full-concentration sample was prepared from one shaded region of Jazzy CBG as described in Section 3.1.4. Serial dilutions of 10^−1^, 10^−2^, 10^−3^, 10^−4^, 10^−5^, 10^−6^, 1/333, and 1/3333 were then created using the full-concentration sample and deionized water. The full-concentration and 10^−1^ dilutions were analyzed in two replicates; all remaining dilutions were analyzed in three replicates. The replicates described for both instruments were pulled from the same dilution tube and run individually.

### 3.3. Exclusivity Study

Twelve non-cannabis materials were selected to represent common cutting agents, herbal products of similar appearance to ground cannabis, the closest botanical relative of cannabis, and human DNA as a potential handling contaminant. These samples included Davidoff tobacco (Davidoff of Geneva USA, Pinellas Park, FL, USA; *Nicotiana tabacum*), rice cereal (*Oryza sativa*), coffee grounds (*Coffea arabica* or *Coffea canephora*), Bigelow green tea (Bigelow Tea Company, Fairfield, CT, USA; *Camellia sinensis*), Ingredients™ chamomile (*Matricaria chamomilla*), oregano (*Origanum vulgare*), Great Value walnuts (Walmart Inc., Bentonville, AR, USA; *Juglans regia*), Thermo Scientific curcumin (Thermo Fisher Scientific, Waltham, MA, USA; a purified compound derived from *Curcuma longa*), nutmeg (*Myristica fragrans*), kratom (*Mitragyna speciosa*), Human (*Homo sapiens*), and Artisan hops (Brewers Supply Group, Shakopee, MN, USA; *Humulus lupulus*).

Human DNA was collected via buccal swab under IRB protocol IRB-FY2017-32804, with informed consent obtained from one donor. For POCKIT Central, one buccal swab was swirled in a 3S tube before two 200 µL aliquots were added into cartridges for analysis. For POCKIT Micro Duo, a second buccal swab was swirled in the sample well of the Grind-N-Go prior to typical downstream analysis.

For POCKIT Central, all remaining non-cannabis materials were prepared in one individual 3S tube, from which two 200 µL replicates were run in individual cartridges. For POCKIT Micro Duo, the remaining non-cannabis materials were prepared in duplicate in two individual Grind-N-Gos, from which 1 µL of extract was used in separate R-tubes. Due to an initial positive result on POCKIT Central, the hops samples were analyzed in four replicates on the POCKIT Central and three on the POCKIT Micro Duo as described above. A negative cannabis result was accepted only where the internal positive control amplified.

### 3.4. Variety Studies

#### 3.4.1. Hemp Variety Study

Thirty-one hemp powder samples sourced from six brands were assessed to evaluate consistency of detection across varieties. Each sample was analyzed once on each platform. Further information about samples, including the brand, type, and strain, can be found in Table A1.

#### 3.4.2. Marijuana Study

Five marijuana samples (*Cannabis sativa* plant material that contains 0.3% delta-9 THC or greater on a dry weight basis) were provided by the National Institute of Standards and Technology (NIST, Gaithersburg, MD, USA). Δ^9^-THC content, reported by NIST as percent mass fraction with uncertainty given as one standard deviation of triplicate measurements, is shown in Table A2. Each sample was analyzed in two replicates taken from the same extraction tube on both platforms.

#### 3.4.3. Hemp Seed Study

Five commercial hemp seed samples were purchased from local grocery stores (Table A3). Following the manufacturer’s instructions for seed samples, one shaded region of the sampling template was used. Because hemp seed is denser than ground flower, this corresponds to approximately 10 mg rather than the approximately 5 mg described in Section 3.1.1 [31]. Each sample was analyzed in a single replicate on each platform.

### 3.5. Interference Study

Tobacco and cannabis were combined at tobacco-to-cannabis ratios of 1×, 10×, 50×, 100×, 200×, and 1000× for the POCKIT Central and 1×, 5×, and 10× for the POCKIT Micro Duo, where *n*× denotes an *n*-fold excess of tobacco relative to cannabis. Ratios were prepared by volume individually for the POCKIT Central and as fractions of the shaded region of the sampling template for the POCKIT Micro Duo. Each ratio was analyzed in two replicates.

For POCKIT Central analysis, cannabis and tobacco were ground in separate 3S Tubes, and defined volumes of each suspension were combined with blank 3S buffer to a constant total of approximately 200 µL before transfer to the cartridge. The volumes used are given in Table 4.

For POCKIT Micro Duo analysis, cannabis and tobacco were apportioned as fractions of the shaded region of the sampling template and ground together in a single Grind-N-Go extraction for each ratio, as shown in Table 5. Two replicates were taken from the single Grind-N-Go.

### 3.6. Statistical Analysis

Detection is reported as the number of positive results out of the number of reactions performed, with the corresponding percentage. Reactions in the sensitivity study were drawn from a single dilution series, and the same dilution tubes were used for both platforms, so template input was matched across instruments. Reactions were performed in duplicate at the full concentration and 10^−1^ dilution and in triplicate at 10^−2^ and below, where results were less consistent; because the number of reactions at the lower dilutions was determined by the results obtained, proportions in the sensitivity study are reported descriptively and no inferential test or confidence interval was calculated for them. In the exclusivity study, proportions were calculated at the material level, with a material counted as positive if any reaction was positive; no interval was calculated for the hops proportion, as the number of replicates run for that material also depended on the initial result. Each material in the variety, marijuana, and hemp seed studies was tested once on each platform, and Wilson score 95% confidence intervals were calculated for the proportion of materials detected in those studies. Analyses were performed in R version 4.6.1. Proportions and Wilson score intervals were computed from the result counts reported in Table 1, Table 2 and Table 3 and Section 2.3. The script used for these calculations is included in the Zenodo deposit cited in the Data Availability Statement, together with the raw instrument outputs from which the counts were derived.

## 4. Conclusions

In this study, an evaluation of iiPCR cannabis assays was conducted using POCKIT Central and POCKIT Micro Duo. The assay was evaluated through sensitivity, exclusivity, and interference studies, and across a range of cannabis material types including hemp varieties, marijuana, and hemp seed. Both the benchtop and portable iiPCR platforms identified cannabis in all cannabis-containing samples tested, supporting their application as rapid screening tools for forensic and field-based analyses. The POCKIT Central was the more sensitive of the two platforms, detecting cannabis DNA at lower template concentrations and across a wider range of tobacco interference than the POCKIT Micro Duo.

All non-cannabis materials tested were negative for cannabis DNA, apart from a single hops replicate on the POCKIT Central, indicating that presumptive positives should be confirmed before reporting. Although the assay does not distinguish between hemp and marijuana, successful screening of cannabis materials can reduce the number of specimens requiring confirmatory analysis, thereby potentially decreasing the forensic evidence backlog over time. Because all testing reported here was performed under controlled laboratory conditions, additional work should extend this assay to cannabis materials encountered at secure screening locations, including airports, seaports, and other controlled-entry facilities. The pre-portioned assay components and straightforward analytical procedure also suggest that these assays could be performed with less specialized training than laboratory-based PCR methods, although operator performance was not assessed in this study. Comparison with existing cannabis screening and identification methods will also be necessary to further validate these platforms. A further limitation is that the assay chemistry is proprietary. Because the primer sequences, premix composition, and reaction parameters are not disclosed by the manufacturer, assay specificity cannot be evaluated in silico; the single reactive hops replicate on the POCKIT Central cannot be attributed to a defined sequence similarity; and independent replication is possible only by acquiring the same commercial platforms and reagent lots rather than by reconstructing the assay from published sequences. Studies of this kind should be read as end-user evaluations of a closed system rather than as characterizations of the underlying assay design.

## Figures and Tables

**Table 1:** Sensitivity study results represented as the number of positive identifications for cannabis out of the number of replicates tested as a fraction and a percent.

Sample	Category	Concentration	POCKIT Central	POCKIT Micro Duo
Standard Concentration Jazzy CBG	Hemp	Full Concentration	2/2 100%	2/2 100%
10^−1^ Concentration Jazzy CBG	Hemp	10^−1^	2/2 100%	2/2 100%
10^−2^ Concentration Jazzy CBG	Hemp	10^−2^	3/3 100%	0/3 0%
10^−3^ Concentration Jazzy CBG	Hemp	10^−3^	3/3 100%	1/3 33.3%
10^−4^ Concentration Jazzy CBG	Hemp	10^−4^	1/3 33.3%	0/3 0%
10^−5^ Concentration Jazzy CBG	Hemp	10^−5^	0/3 0%	0/3 0%
10^−6^ Concentration Jazzy CBG	Hemp	10^−6^	0/3 0%	0/3 0%
$\frac{1}{333}$ Concentration Jazzy CBG	Hemp	$\frac{1}{333}$	3/3 100%	2/3 66.7%
$\frac{1}{3333}$ Concentration Jazzy CBG	Hemp	$\frac{1}{3333}$	3/3 100%	1/3 33.3%

**Table 2:** Exclusivity study results represented as the number of positive identifications for cannabis out of the number of replicates tested, as a fraction and a percent.

Sample	Category	Concentration	POCKIT Central	POCKIT Micro Duo
Tobacco	Non-cannabis material	Full Concentration	0/2 0%	0/2 0%
Rice Cereal	Non-cannabis material	Full Concentration	0/2 0%	0/2 0%
Coffee Grounds	Non-cannabis material	Full Concentration	0/2 0%	0/2 0%
Green Tea	Non-cannabis material	Full Concentration	0/2 0%	0/2 0%
Chamomile	Non-cannabis material	Full Concentration	0/2 0%	0/2 0%
Oregano	Non-cannabis material	Full Concentration	0/2 0%	0/2 0%
Walnuts	Non-cannabis material	Full Concentration	0/2 0%	0/2 0%
Curcumin	Non-cannabis material	Full Concentration	0/2 0%	0/2 0%
Nutmeg	Non-cannabis material	Full Concentration	0/2 0%	0/2 0%
Kratom	Non-cannabis material	Full Concentration	0/2 0%	0/2 0%
Human	Non-cannabis material	Full Concentration	0/2 0%	0/2 0%
Hops	Non-cannabis material	Full Concentration	1/4 25%	0/3 0%

**Table 3:** Interference study results represented as the number of positive identifications for cannabis out of the number of replicates tested as a fraction and a percent.

Sample	Category	POCKIT Central	POCKIT Micro Duo
1×	Hemp and Tobacco	2/2 100%	2/2 100%
5×	Hemp and Tobacco	NA	2/2 100%
10×	Hemp and Tobacco	2/2 100%	2/2 100%
50×	Hemp and Tobacco	2/2 100%	NA
100×	Hemp and Tobacco	2/2 100%	NA
200×	Hemp and Tobacco	2/2 100%	NA
1000×	Hemp and Tobacco	2/2 100%	NA

**Table 4:** POCKIT Central sample preparation for cannabis and tobacco ratios.

	Cannabis	Tobacco	Blank 3S
1×	0.2 µL	0.2 µL	200 µL
10×	0.2 µL	2 µL	198 µL
50×	0.2 µL	10 µL	190 µL
100×	0.2 µL	20 µL	180 µL
200×	0.2 µL	40 µL	160 µL
1000×	0.2 µL	200 µL	0 µL

**Table 5:** POCKIT Micro Duo sample preparation for cannabis and tobacco ratios.

	Cannabis	Tobacco
1×	$\frac{1}{2}\;Shaded\;area$	$\frac{1}{2}\;Shaded\;area$
5×	$\frac{1}{5}\;Shaded\;area$	$\frac{4}{5}\;Shaded\;area$
10×	$\frac{1}{10}\;Shaded\;area$	$\frac{9}{10}\;Shaded\;area$

## Data Availability

All data generated in this study are provided within the article and the appendix. The underlying raw instrument outputs, comprising the exported run files and per-reaction result reports for every reaction described, have been deposited in Zenodo and are publicly available at https://doi.org/10.5281/zenodo.22874792. The primer sequences, premix composition, internal control identity, and reaction parameters of the commercial assay are proprietary to GeneReach Biotechnology Corporation, were not available to the authors, and cannot be supplied by them; enquiries regarding those parameters should be directed to the manufacturer.

## Appendix A

### Sample Information and Preparation

**Table A1:** Hemp sample source, type, and strain information. Suppliers: CBD Hemp Direct (Las Vegas, NV, USA), Industrial Hemp Farms (Colorado Springs, CO, USA), The Original Hemp Buds (Los Angeles, CA, USA), ATLRx (Alpharetta, GA, USA), Green Unicorn Farms (San Rafael, CA, USA), and CBD Supply Maryland (Baltimore, MD, USA).

Source/Brand	Type	Strain
CBD Hemp Direct	CBD Flower	Paradise OG
CBD Hemp Direct	CBD Hemp	Juicy Fruit #2
Industrial Hemp Farms	CBD Hemp	Bubba Merlot
CBD Hemp Direct	CBD Hemp	Sunset Road Sherbert #2
CBD Hemp Direct	CBD Hemp	Trophy Wife
CBD Hemp Direct	CBD Hemp	Casino Cookies #2
CBD Hemp Direct	CBD Hemp	Tangie
Industrial Hemp Farms	CBD Hemp	Pineapple Kush
CBD Hemp Direct	CBD Hemp	Hemp World Haze
The Original Hemp Buds	CBD Hemp	Special Sauce
The Original Hemp Buds	CBD Hemp	Electra
The Original Hemp Buds	CBD Hemp	Lifter
The Original Hemp Buds	CBD Hemp	Sour Space Candy
The Original Hemp Buds	CBD Hemp	Cascade
Industrial Hemp Farms	CBD Hemp	Magic Jordan CBG
ATLRx Premium CBD flower	Δ8 Hemp	Candyland
ATLRx Premium CBD flower	Δ8 Hemp	Fruity Pebbles
ATLRx Premium CBD flower	Δ8 Hemp	Northern Lights
ATLRx Premium CBD flower	Δ8 Hemp	Sour Jack
ATLRx Premium CBD flower	Δ8 Hemp	White Cap
ATLRx Premium CBD flower	Δ8 Hemp	Cherry Runtz
ATLRx Premium CBD flower	Δ8 Hemp	THC-O Lemon Haze
ATLRx Premium CBD flower	Δ8 Hemp	THC-O Suver Haze
ATLRx Premium CBD flower	Δ8 Hemp	D10 Chemdawg OG
ATLRx Premium CBD flower	Δ8 Hemp	Indoor CBG Sour
ATLRx Premium CBD flower	Δ8 Hemp	Indoor CBG White
Green Unicorn Farms	CBDV Hemp	Pine Walker
CBD Hemp Direct	CBG Hemp	Desert Snow CBG
Industrial Hemp Farms	CBG Hemp	Jack Frost CBG
CBD Supply Maryland	Delta 8 Cookies infused hemp flower	Delta 8 Cookies infused hemp flower
CBD Supply Maryland	Delta 8 Moonrocks CBD Flower + delta8 oil and kief	Limeade Fuel

**Table A2:** Δ^9^-THC of marijuana samples.

Sample	Δ^9^-THC Content
MJ1	4.41 ± 1.10
MJ2	5.02 ± 1.26
MJ3	6.54 ± 1.64
MJ4	3.61 ± 0.90
MJ5	5.46 ± 1.37

**Table A3:** Hemp seed sample information. Suppliers: Manitoba Harvest (Winnipeg, MB, Canada), Badia Spices (Doral, FL, USA), Navitas Organics (Novato, CA, USA), and American Hemp Harvest (Boulder, CO, USA).

Hemp Seed Sample	Brand Information
Hemp Seed 1	Manitoba Harvest Hemp Hearts
Hemp Seed 2	Badia Hulled Hemp Seeds
Hemp Seed 3	Navitas Organics
Hemp Seed 4	Manitoba Harvest Hemp Hearts
Hemp Seed 4	American Hemp Harvest Hemp Hearts
